# The Prognostic Significance of Beta2 Microglobulin in Patients with Hemophagocytic Lymphohistiocytosis

**DOI:** 10.1155/2016/1523959

**Published:** 2016-03-27

**Authors:** Tiantong Jiang, Xiurong Ding, Weixing Lu

**Affiliations:** ^1^Department of Cardiology, Beijing University of Chinese Medicine Third Affiliated Hospital, Beijing 100029, China; ^2^Departments of Clinical Laboratory, Beijing Youan Hospital, Capital Medical University, Beijing 100069, China

## Abstract

*Objective*. To determine the prognostic significance of beta2 microglobulin (*β*_2_-m) concentrations in patients with hemophagocytic lymphohistiocytosis (HLH), a rare disorder caused by pathologic activation of the immune system.* Patients and Methods*. The study population consisted of 74 patients diagnosed with HLH and 35 healthy controls. Serum *β*_2_-m levels were measured using a latex agglutination photometric immunoassay.* Results*. Median serum *β*_2_-m levels were significantly higher in HLH patients than in healthy controls (4.05 versus 1.5 mg/L; *P* < 0.001) and were significantly higher in patients with lymphoma associated hemophagocytic syndrome (LAHS) than in patients with benign disease-associated HLH (4.2 versus 3.3 mg/L; *P* < 0.001). Higher serum *β*_2_-m levels were positively correlated with LAHS (*P* = 0.005), abnormal lactate dehydrogenase concentrations (*P* = 0.009), and hypoalbuminemia (*P* = 0.003). ROC analysis showed that overall survival (OS) was significantly shorter in LAHS patients with serum *β*_2_-m levels ≥4.03 mg/L compared to <4.03 mg/L (*P* < 0.001). Moreover, multivariate analysis showed that serum *β*_2_-m level was an independent prognostic of OS (*P* = 0.034) in patients with LAHS.* Conclusion*. High serum *β*_2_-m levels and LAHS were associated with markedly poorer OS in patients with HLH. Serum *β*_2_-m concentration was a powerful and independent prognostic factor for OS in patients with LAHS.

## 1. Introduction

Hemophagocytic lymphohistiocytosis (HLH), also known as hemophagocytic syndrome, is an uncommon systemic inflammatory clinical syndrome characterized by the increased proliferation of benign macrophages, which phagocytose blood cells throughout the reticuloendothelial system [1]. HLH has been traditionally divided into a primary form, which typically manifests in children with documented genetic abnormalities of the cytotoxic functions of NK cells and T cells, and a secondary form that tends to occur at older ages in the setting of an associated condition, such as infection, malignancy, or autoimmune disease, without an identifiable genetic abnormality [2]. Although the pathogenesis of secondary HLH is not as well understood as that of primary HLH, infection associated HLH and lymphoma associated hemophagocytic syndrome (LAHS) are generally recognized as the two most common forms of secondary HLH [3]. LAHS has been reported to account for approximately 40% of adult-onset secondary HLH [4], with an incidence of 0.36 per 100,000 adults per year [5]. Despite advances in therapy, the prognosis of HLH is poor, with 40–60% of patients initially unresponsive to treatment and dying of HLH, infections, or complications during therapy [3]. Few reports, however, have assessed factors prognostic of survival in patients with HLH.

*β*_2_ microglobulin (*β*_2_-m) is a low-molecular-weight protein synthesized in all nucleated cells and constituting the light chain subunit of major histocompatibility complex (MHC) class I antigens. Under physiological conditions, *β*_2_-m is generated at a constant rate, except in patients with systemic inflammation or hematopoietic neoplasia, such as multiple myeloma, B cell chronic lymphocytic leukemia, and Hodgkin's lymphoma. Moreover, serum *β*_2_-m concentration has been shown to be independently prognostic for these diseases [6–8], as well as an independent predictor of total mortality in a general population of older adults [9]. Patients in our institution with a confirmed diagnosis of secondary HLH had high serum *β*_2_-m levels, in agreement with previous findings [10], suggesting that serum *β*_2_-m levels may have prognostic significance in these patients. This study therefore evaluated whether serum *β*_2_-m concentration is a prognostic factor in patients with secondary HLH.

## 2. Methods

### 2.1. Patients and Methods

Between November 2012 and May 2014, 81 patients admitted to Beijing Friendship Hospital, Beijing University of Chinese Medicine Third Affiliated Hospital, and Beijing Youan Hospital were diagnosed with HLH according to the HLH-2004 diagnostic guidelines [11]. These 81 patients included three with primary and 78 patients with secondary HLH. Malignant lymphoma was diagnosed according to the 2008 World Health Organization criteria [12]. Of the 78 patients with secondary HLH, four had incomplete data and were excluded. Complete data were obtained for 74 patients, including age; presumed etiology of HLH; presence or absence of splenomegaly; white blood cell (WBC) and platelet (PLT) counts; concentrations of hemoglobin (Hb), alanine aminotransferase (ALT), aspartate aminotransferase (AST), lactate dehydrogenase (LDH), triglycerides (TG), fibrinogen (Fib), and ferritin; and hemophagocytosis in the bone marrow. The healthy control group consisted of 35 subjects, of median age 36.5 years (range 21–54 years) undergoing routine medical examinations at the same hospital. Serum concentrations of *β*_2_-m (normal range 1.0–1.9 mg/L) were measured using a latex agglutination photometric immunoassay (Eiken Chemicals, Tokyo, Japan). This study was approved by the Medical Ethics Committee of Beijing University of Chinese Medicine Third Affiliated Hospital, Beijing, China, which waived the requirement for individual informed consent.

### 2.2. Statistical Analysis

Categorical variables were reported as proportion and continuous variables as medians and 25th–75th and 5th–95th percentiles, as appropriate. Overall survival (OS) was defined as the time between the first day of diagnosis and the date of death from any cause or last follow-up. OS rates were estimated by the Kaplan-Meier method and compared using the log-rank test. Multivariate prognostic analyses for OS were performed using Cox proportional hazards regression model with backward (conditional) elimination. ROC curve analysis was used to determine the optimal cutoff point for *β*_2_-m concentration; the concentration with maximum sensitivity and specificity was selected. The binary clinical outcome (death/survival) was determined six months after diagnosis. Patients were categorized as “alive/censored” when the follow-up time was longer than six months and “dead” if the patient had died before this time point. Categorical variables were compared using *χ* ^2^ tests and continuous variables were compared using Mann-Whitney *U* tests. All statistical analyses were performed using the SPSS version 17.0 statistical software program (SPSS, Chicago, IL), with a *P* value less than 0.05 considered statistically significant.

## 3. Results

### 3.1. Clinical Features

The baseline characteristics of the 74 patients with secondary HLH are shown in Table 1. According to presumed etiology, patients were divided into two subgroups, an LAHS of 41 patients and a benign disease-associated HLH group of 33 patients. The most frequent histopathological subtype in the LAHS group was extranodal NK/T cell lymphoma (ENKL; *n* = 16), followed by peripheral T cell lymphoma (PTCL; *n* = 11), diffuse large B cell lymphoma (DLBCL), not otherwise specified (NOS; *n* = 10), chronic lymphocytic leukemia (CLL; *n* = 2), *γδ* T cell lymphoma (*γδ*-TCL; *n* = 1), and subcutaneous panniculitis like T cell lymphoma (SPTCL; *n* = 1).

Of the 33 patients with benign disease-associated HLH, 24 had infectious etiology, including infection with Epstein-Barr virus (EBV; *n* = 18), cytomegalovirus (CMV; *n* = 2); pneumonia (*n* = 3); and tuberculosis (*n* = 1). In 4 patients, HLH was associated with autoimmune disorders, including adult-onset Still's disease (AOSD; *n* = 2), sicca syndrome (SS; *n* = 1), and undifferentiated connective tissue disease (UCTD; *n* = 1), whereas, in one patient, HLH was associated with acute fatty liver of pregnancy (AFLP). The underlying disease could not be determined in four patients despite testing for EBV, CMV, parvovirus, sepsis, autoimmune disorders, and malignancy.

During follow-up, 43/74 (58.1%) patients died, 26/41 (63.4%) in the LAHS group and 17/33 (51.5%) in the group with benign disease-associated HLH. Median overall survival was 4 months.

### 3.2. Serum *β*_2_-m Levels

**Figure 1:**
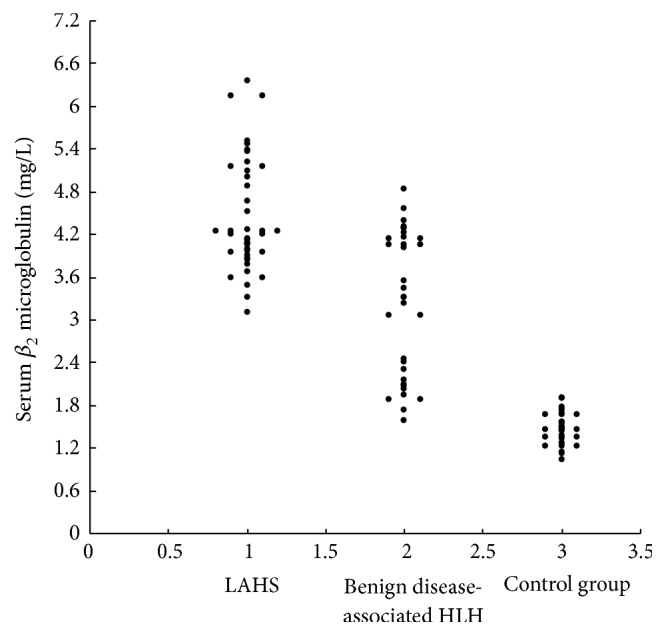
Serum *β*_2_-m concentrations in the 41 patients with lymphoma associated hemophagocytic syndrome (LAHS), the 33 patients with benign disease-associated hemophagocytic lymphohistiocytosis (HLH) and 35 controls.

The median serum *β*_2_-m concentration in all 74 patients with HLH was 4.05 mg/L (5th–95th percentile range, 1.9 to 5.7 mg/L), significantly higher than that in the 35 normal volunteers (median, 1.5 mg/L; 5th–95th percentile range 1.1–1.9 mg/L; *P* < 0.001) (Figure 1). Assessments in patient subgroups showed that the median serum *β*_2_-m concentration was significantly higher in the LAHS (4.2 mg/L; 5th–95th percentile range 3.3–6.2 mg/L) than in the benign disease-associated HLH (3.3 mg/L, 5th–95th percentile range 1.7–4.6 mg/L) group (*P* < 0.001). Serum *β*_2_-m levels were also significantly higher in each of these subgroups than in the normal control group (*P* < 0.001 each).

### 3.3. Determining the Cutoff Value of Serum *β*_2_-m for OS

**Figure 2:**
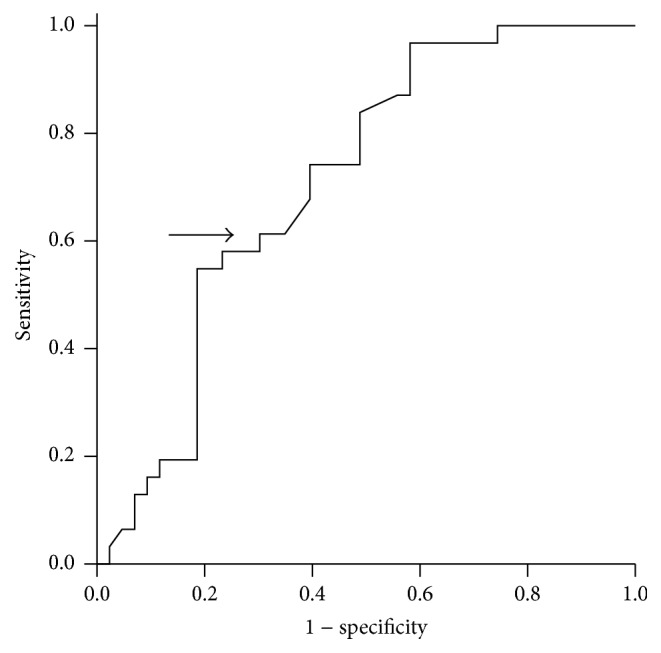
Receiver operating characteristic and area under the curve (AUC) analysis of the serum *β*_2_-m levels at the diagnosis of HLH (AUC = 0.71; 95% confidence interval = 0.592–0.827).

An ROC curve of OS as a function of serum *β*_2_-m concentration was generated to determine the optimum cutoff value of the latter [13]. The area under the curve was recorded 0.71 (95% confidence interval [CI], 0.592–0.827; Figure 2). A serum *β*_2_-m concentration of 4.03 mg/L yielded maximum combined sensitivity (62%) and specificity (65%) on the ROC curve.

### 3.4. Correlation of Serum *β*_2_-m with Other Laboratory Variables

To evaluate the relevance of serum *β*_2_-m ≥4.03 mg/L at diagnosis in patients with HLH, patients were divided according to this cutoff. Of the 74 patients, 36 (48.6%) had serum *β*_2_-m levels <4.03 mg/L and 38 (51.4%) had serum *β*_2_-m levels ≥4.03 mg/L. Patients with serum *β*_2_-m ≥4.03 mg/L at diagnosis were significantly more likely to present with LAHS (*P* = 0.005), abnormal LDH (*P* = 0.009), and hypoalbuminemia (*P* = 0.003) than those with serum *β*_2_-m <4.03 mg/L. The two groups did not differ significantly in age (*P* = 0.642), sex (*P* = 0.506), abnormal WBC count (*P* = 0.924), medium or severe anemia (*P* = 0.598), thrombocytopenia (*P* = 0.367), hypofibrinogenemia (*P* = 0.839), hypertriglyceridemia (*P* = 0.872), abnormal ALT (*P* = 0.255), abnormal AST (*P* = 0.511), or significantly increased ferritin (<6000 ng/mL versus ≥6000 ng/mL, *P* = 0.163).

### 3.5. Prognostic Significance of Serum *β*_2_-m Concentration

**Figure 3:**
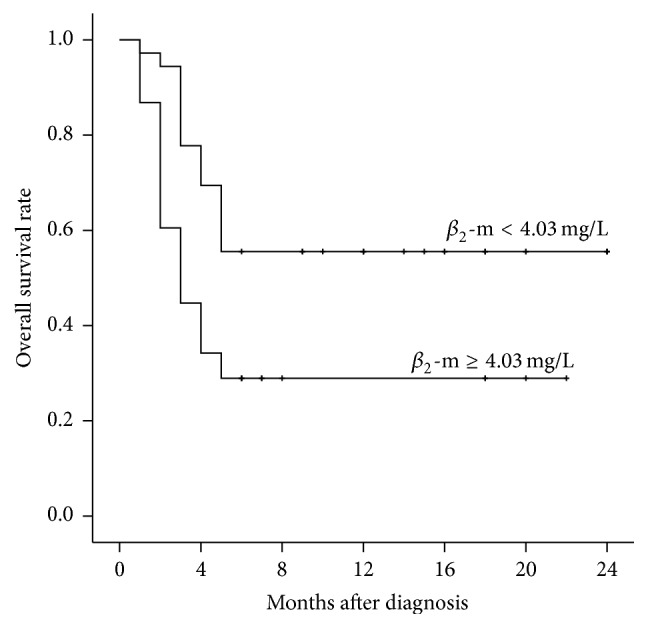
Kaplan-Meier analysis of overall survival rates in patients with serum *β*_2_-m concentrations <4.03 mg/L and ≥4.03 mg/L at diagnosis (*P* = 0.003).

**Figure 4:**
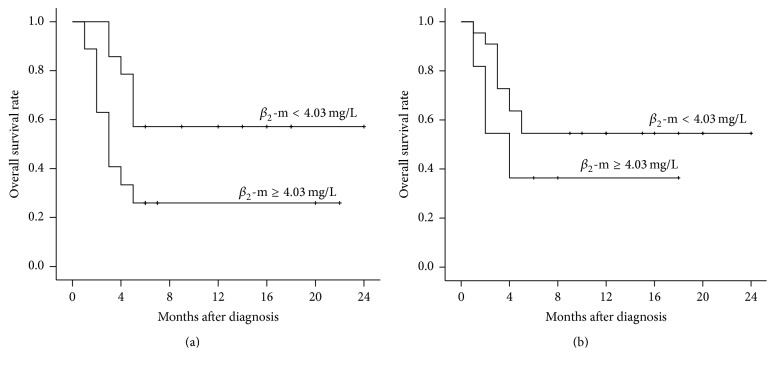
Kaplan-Meier analyses of overall survival in the subgroups of patients with LAHS (a) and benign disease-associated HLH (b) and serum *β*_2_-m concentrations <4.03 mg/L and ≥4.03 mg/L at diagnosis ((a) *P* = 0.015; (b) *P* = 0.177).

Six-month OS rates were significantly lower in patients with serum *β*_2_-m ≥4.03 mg/L compared to <4.03 mg/L (28.9% versus 55.6%; *P* < 0.001; Figure 3). Similarly, six-month OS rates in the LAHS subgroup were significantly lower in patients with higher serum *β*_2_-m levels (*P* = 0.015, Figure 4(a)). Six-month OS rates were also lower in patients with benign disease-associated HLH with higher serum *β*_2_-m, but this difference was not statistically significant (*P* = 0.177, Figure 4(b)).

Univariate analysis showed that OS correlated significantly with thrombocytopenia (<40 × 10^9^/L), hypoalbuminemia (<30 g/L), abnormal LDH (≥1000 U/L), and serum *β*_2_-m concentration ≥4.03 mg/L (Table 2). This makes *β*_2_-m levels a useful diagnostic marker for HLH. Multivariate analysis adjusting for lymphoma, thrombocytopenia, hypoalbuminemia, abnormal LDH, and serum *β*_2_-m concentration ≥4.03 mg/L showed that serum *β*_2_-m level ≥4.03 mg/L (*P* = 0.030) and thrombocytopenia (*P* = 0.037) were independent prognostic markers of reduced OS (Table 2). Analysis of these variables in patient subgroups showed that serum *β*_2_-m ≥4.03 mg/L remained independently prognostic in patients with LAHS (*P* = 0.034), but not in patients with benign disease-associated HLH (*P* = 0.102).

## 4. Discussion

HLH is a clinical syndrome, in which an exaggerated inflammatory reaction is triggered by various inherited and/or acquired factors [2]. Even when treated in a timely manner, all forms of HLH can be fatal. Malignancy-associated HLH has a poorer prognosis than other forms of HLH [14]. This study found that serum *β*_2_-m levels were elevated in all patients with HLH patients, whereas their serum creatinine levels were within the normal range (data not shown). Moreover, serum *β*_2_-m level was an independent predictor of OS in patients with LAHS.

The protein *β*_2_-m consists of a single polypeptide chain, which is linked noncovalently to MHC class I cell surface antigens. Membrane turnover is the principal source of *β*_2_-m in the blood. This protein has been reported to be involved in cell survival, proliferation, and metastasis in various types of cancer, with serum *β*_2_-m level being directly related to tumor burden [15, 16]. Elevated levels of serum *β*_2_-m have been reported in various diseases. For example, 47% of patients with extranodal natural killer (NK)/T cell lymphoma were reported to have elevated serum *β*_2_-m concentrations [17]. Moreover, serum *β*_2_-m levels have been associated with the malignancy of lymphoma [18] and elevated in 40–55% of patients with aggressive non-Hodgkin's lymphoma [19, 20], with the proportion of patients having elevated serum *β*_2_-m varying by the primary site of diseases [21]. In addition, serum *β*_2_-m concentration has been reported to be a predictor of clinical outcome, prognosis, and tumor burden in patients with various types of lymphomas [7, 8, 22].

This study found that serum *β*_2_-m concentrations were elevated in almost all patients with HLH, especially those with LAHS. However, we found no cutoff with sufficient specificity and sensitivity to distinguish LAHS from benign disease-associated HLH (data not shown). However, both univariate and multivariate analyses revealed that elevated serum *β*_2_-m levels were an adverse prognostic factor in patients with LAHS, in agreement with previous results in lymphoma patients [7, 8].

The biological basis underlying the potential adverse prognostic significance of elevated serum *β*_2_-m remains unclear. In diffuse large cell lymphoma, the absence of MHC class I expression correlated with higher serum *β*_2_-m levels [23]. These patients have a particularly poor prognosis, presumably because of defective recognition of tumor-specific antigens by cytotoxic T cells [24]. Other studies have reported that *β*_2_-m induces the apoptosis of neoplastic T cells and myeloid leukemic cells and may regulate the elimination of tumor cells [25, 26]. Additional studies are needed to determine whether *β*_2_-m is only a prognostic factor in patients with lymphoma and related diseases, such as LASH, or whether its increase is associated with the killing of tumor cells.

Assessment of other laboratory markers showed that only thrombocytopenia was an independent prognostic marker for survival in patients with HLH, similar to previous findings [27]. Although a recent study showed that hypoalbuminemia was a significant predictor of poorer inferior survival of HLH patients on multivariate analysis [14], our study found a significant correlation between hypoalbuminemia and OS on univariate, but not multivariate, analysis. This may be explained by the difference in study population, in that the earlier study included only patients aged ≥18 years, whereas 16% (12/74) of our patients were <18 years. Moreover, differences in the underlying diseases of these patients likely affected outcomes.

In summary, this study found that a higher baseline concentration of serum *β*_2_-m was a powerful predictor of mortality in patients with LAHS. These findings suggest the need for large clinical trials assessing the significance of serum *β*_2_-m concentrations in patients with LAHS.

## Figures and Tables

**Table 1:** Baseline demographic and clinical characteristics of patients with HLH.

	Total	LAHS	Benign disease-associated HLH
	*n* = 74	*n* = 41	*n* = 33
Age, yr	39.0 (22.3–54.3)	44.0 (24.5–54.0)	31.0 (19.0–55.0)
Male, number (%)	44 (59.5%)	28 (68.3%)	16 (48.5%)
Fever (>38°C)	73 (98.6%)	41 (100%)	32 (97.0%)
Splenomegaly	63 (85.1%)	36 (87.8%)	27 (81.8%)
Leukocytes, ×10^9^/L	2.7 (1.2–4.0)	2.6 (1.1–3.7)	3.2 (1.5–4.5)
Hemoglobin, g/L	86.0 (76.0–103.8)	86.0 (72.5–105.0)	86.0 (77.0–102.0)
Platelets, ×10^9^/L	36.9 (25.0–66.3)	36.7 (21.0–83.0)	39.0 (25.9–56.0)
Triglycerides, mmol/L	2.5 (1.6–3.6)	2.7 (2.1–3.8)	2.1 (1.5–3.5)
Fibrinogen, g/L	1.5 (0.9–2.6)	1.3 (0.9–2.6)	1.7 (1.1–2.6)
Ferritin, *μ*g/L	6268.5 (1975.0–14802.5)	6500.0 (1825.5–15000)	5973.0 (2023.5–14734.5)
NK cell activity (%)	12.4 (9.6–16.3)	11.7 (8.4–17.1)	12.7 (11.4–16.0)
sCD25 ≥ 2400 U/mL	70 (94.6%)	40 (97.6%)	30 (90.9%)
Hemophagocytosis	61 (82.4%)	32 (78.0%)	29 (87.9%)

Results reported as median (range) or number (%).

**Table 2:** Univariate and multivariate analysis of factors associated with OS in patients with HLH.

Covariate			OS	
		HR	95% CI	*P* value
		Univariate analysis		
Age, y	<40 versus ≥40	1.164	0.639–2.120	0.620
WBC (×10^9^/L)	<4 versus ≥4	0.518	0.248–1.082	0.080
Hb (g/L)	<90 versus ≥90	0.722	0.381–1.367	0.317
PLT (×10^9^/L)	<40 versus ≥40	0.512	0.270–0.970	0.040
TG (mmol/L)	<3 versus ≥3	1.726	0.938–3.177	0.080
Fib (g/L)	<1.5 versus ≥1.5	0.791	0.435–1.441	0.444
Ferritin (ng/mL)	<6000 versus ≥6000	1.673	0.912–3.072	0.097
ALT (U/L)	<40 versus ≥40	1.129	0.613–2.081	0.697
AST (U/L)	<40 versus ≥40	1.184	0.617–2.270	0.611
ALB (g/L)	<30 versus ≥30	0.507	0.270–0.951	0.034
LDH (U/L)	<1000 versus ≥1000	1.902	1.043–3.470	0.036
β_2_-m (mg/L)	<4.03 versus ≥4.03	2.306	1.237–4.298	0.009

		Multivariate analysis		
PLT (×10^9^/L)	<40 versus ≥40	0.502	0.262–0.959	0.037
ALB (g/L)	<30 versus ≥30	0.739	0.347–1.572	0.432
LDH (U/L)	<1000 versus ≥1000	1.309	0.643–2.664	0.458
*β*_2_-m (mg/L)	<4.03 versus ≥4.03	2.129	1.076–4.213	0.030
Lymphoma	(+) versus (−)	0.850	0.434–1.666	0.636

WBC, white blood cell; Hb, hemoglobin; PLT, platelet; TG, triglyceride; Fib, fibrinogen; ALT, alanine aminotransferase; AST, aspartate aminotransferase; ALB, albumin; LDH, lactic dehydrogenase; and β_2_-m, beta2 microglobulin. The detection limit of ferritin was 15000 ng/mL; patients with ferritin concentrations higher than this upper limit were assigned a value of 15000 ng/mL for univariate and multivariate analyses.
